# Conversation Barriers and Strategies Used by People With Parkinson's and Their Partners to Support Conversation

**DOI:** 10.1111/1460-6984.70202

**Published:** 2026-01-25

**Authors:** Ramishka Thilakaratne, Karen Wylie, Andrea M. Loftus, Naomi Cocks

**Affiliations:** ^1^ Curtin School of Allied Health Curtin University Perth Western Australia Australia; ^2^ School of Biomedical and Allied Health Sciences University of Ghana Accra Ghana; ^3^ ParkC, Curtin University Perth Western Australia Australia

**Keywords:** communication partner, conversation, lived experience, Parkinson's disease

## Abstract

**Background:**

People with Parkinson's experience a range of communication difficulties impacting their conversations. As conversations are a two‐way or more interaction, communication partners play an important role in conversational success.

**Aims:**

This qualitative exploratory study sought to capture the lived experience of people with Parkinson's and their communication partners regarding (i) their perceptions of barriers to successful conversations and (ii) strategies perceived as helpful to support their conversations.

**Methods and Procedures:**

Data were collected from 45 participants (25 people with Parkinson's and 20 communication partners) across five focus groups and analysed using qualitative content analysis.

**Outcomes and Results:**

Participants discussed five categories of barriers to conversation. These related to the person with Parkinson's, communication partner, conversation dynamic, background noise, and limited information and services. Participants described six categories of strategies used to support conversations. These included strategies used to prepare for conversations, during conversations, for phone use, acceptance and awareness, services and information, and engagement in activities.

**Conclusions and Implications:**

People with Parkinson's face unique challenges in conversation and may benefit from a range of strategies to support conversational success. Through their lived experience of Parkinson's, they have developed strategies that support conversational success. Both the person with Parkinson's and their partner contribute to the success of a conversation. New findings revealed the impact of cognitive load on conversations. These findings warrant careful consideration in the development of future communication‐focused therapeutic approaches for individuals with Parkinson's and their families.

**WHAT THIS PAPER ADDS:**

*What is already known on the subject*

- Parkinson's disease is a motor speech disorder which not only impacts the person physically but also impacts their communication and social interaction thereby impacting their quality of life. Difficulty engaging in conversations is a common challenge reported by people with Parkinson's. Through their lived experience, people with Parkinson's and their partners have understood the common barriers they face in conversations and have developed a range of strategies to support their conversations with each other.

*What this paper adds to existing knowledge*

- Participants in this study described their lived experience of Parkinson's and shared the strategies they used to support conversations. These included strategies that could be used in preparation for and during conversations, with phone use, strategies related to acceptance and awareness of the condition and accessing services and information. This work highlights the important role played by both the person with Parkinson's and their communication partner in promoting conversational success to support their quality of life.

*What are the potential or actual clinical implications of this work?*

- This study provides clear implications for therapy supporting conversation in this population. It also highlights the need for future research assessing the effectiveness of conversation strategies that people with Parkinson's and their communication partners perceive to be effective.

## 1 Introduction

Parkinson's Disease (Parkinson's) is one of the most common neurodegenerative disorders (Pringsheim et al. 2014). Up to 90% of people with Parkinson's develop communication difficulties, ranging in presentation and severity (Miller 2017; Miller et al. 2006; Smith and Caplan 2018). Communication difficulties include reduced speech intelligibility, reduced volume of voice, word finding difficulties (Schalling et al. 2017), difficulties with social communication (Hall et al. 2011), and a combination of these difficulties (Griffiths et al. 2015; Whitehead 2010). These difficulties impact the ability of the person with Parkinson's to effectively and enjoyably engage in conversations (Griffiths et al. 2015).

Conversations are communication between two or more people, enabling a person to actively participate in social situations (Turnbull 2003). Conversations are essential to developing and maintaining relationships (Francis and Hester 2004). Conversations are significantly impacted when one participant has a communication impairment (Eriksson et al. 2016). Being unable to converse effectively with a communication partner (the other person participating in the conversation) adversely impacts quality of life for both (Griffiths et al. 2011), and creates embarrassment and social withdrawal (Moya‐Galé et al. 2025).

People with Parkinson's and their partners report that they struggle to have meaningful conversations (Holtgraves and Cadle 2016). An inability to follow and maintain a topic of conversation and speech issues (including reduced volume, inarticulation) have been identified as barriers to successful conversations (Schalling et al. 2017). Despite conversational difficulties being a key concern in Parkinson's, current communication treatments focus on improving speech or providing compensatory speaker‐oriented techniques (Griffiths et al. 2011). A review of communication strategies used by speech pathologists in Parkinson's revealed that voice quality and speech intelligibility was typically targeted (Griffiths et al. 2011). Despite being the primary reason for referral to speech services, difficulties relating to word‐finding, following a conversation, and keeping on‐topic, were rarely considered (Griffiths et al. 2011).

All individuals involved in a conversation contribute to maintaining the conversation (Eriksson et al. 2016; Hadley et al. 2019; Thilakaratne et al. 2021). The communication partner plays a key role, as they often need to modify their communication when conversation breakdown occurs (Eriksson et al. 2016; Thilakaratne et al. 2021). Due to the complex nature of communication difficulties experienced by people with Parkinson's (Holtgraves and Cadle 2016), they often depend on their communication partner's support to maintain a conversation (Saldert and Bauer 2017). While interventions that are currently used can promote communicative outcomes for people with Parkinson's, there is a need for more evidence‐based interventions that address interaction and engagement (Baylor et al. 2024). Therapy approaches that target conversational success must therefore consider the input and participation of all communication partners involved (Bloch and Beeke 2008; Whitworth et al. 1999).

A scoping review of conversation interventions for people with Parkinson's found that only one study (Forsgren et al. 2013) considered the important dynamic between the person with Parkinson's and their communication partner (Thilakaratne et al. 2021). Conversations between the person with Parkinson's and their communication partner were recorded and analysed to identify specific difficulties they experienced. The dyad was then taught relevant strategies to address those specific difficulties and develop more successful conversations (Forsgren et al. 2013). Encouragingly, some other planned interventions will focus on communication as an intervention process for people with Parkinson's (Clay et al. 2023). For example, a recently published protocol for the work of ‘Better Conversations with Parkinson's’ states that conversation analysis will be used to provide feedback on specific skills that can improve conversations in dyads (Clay et al. 2023).

A substantial body of research has explored the barriers and facilitators to successful conversation for people with other types of communication difficulties and their partners, such as aphasia (Simmons‐Mackie et al. 2016). However, the symptoms and treatment of Parkinson's differ from those of other types of communication difficulties. Due to the specific communication difficulties associated with Parkinson's, the impact of neurodegeneration, and the impact of medications, findings relating to communication in other conditions are not always directly applicable (Hayes 2019). The unique communication challenges faced by people with Parkinson's have led to the development of specific strategies to support their interactions. Experiential knowledge about what has or has not worked for them in everyday communication is invaluable to the development of these strategies. This is a form of practice‐based evidence that could be used to support others facing similar communication challenges (Videmšek and Fox 2018).

To date, four qualitative studies have focussed on the barriers and facilitators to conversations for people with Parkinson's and their close communication partners (Johansson et al. 2020; Miller et al. 2006; Whitehead 2010; Wylie et al. 2022), with less exploration of conversation facilitators. Whitehead (2010) and Johansson et al. (2020) interviewed four and six people, respectively, with Parkinson's and their communication partners about the communication changes they had experienced. Whitehead (2010) described the impact of Parkinson's on the person and their partner while Johansson et al. (2020) highlighted the barriers to conversations with some information on facilitators. Participant numbers in these studies were low, with Whitehead (2010) and Johansson et al. (2020) interviewing four and six people, respectively about the communication changes they had experienced. Miller et al. (2006) interviewed 37 people with Parkinson's about conversation difficulties, but they did not include communication partners. Participants described the impact of Parkinson's on their speech and voice, along with strategies they used to address these challenges, which were largely focussed on general strategies enacted by the person with Parkinson's, such as being passive in conversation. The most recent study, Wylie et al. (2022), interviewed eight people with Parkinson's and their communication partners, focussing on both the barriers to and facilitators of conversations. Their findings moved beyond those of Miller et al. (2006). They described conversation being influenced not only by the communication skills of the person with Parkinson's, but also by factors relating to the communication partner, the complex nature of conversations, the communication environment, and the impact of experience in shaping participation in conversation (Wylie et al. 2022). This study also identified several facilitators of effective conversations, including specific strategies used and the importance of knowledge about Parkinson's. It was evident that, through their lived experience, many people with Parkinson's and their partners had developed considerable knowledge and strategies that could be shared to support others to have more meaningful conversations.

Whilst the above‐mentioned studies contribute to our understanding of the impact of Parkinson's on communication, there is a need to further extend understanding of the barriers and particularly the facilitators to conversations in greater depth with a wider range of participants and by using other methodologies. Expanding understanding of the lived experiences and perspectives of both the person with Parkinson's and their communication partners on what they have found to be effective in supporting conversations will build the evidence base around strategies to support conversations. Building this lived‐experience evidence base could potentially support the development of a clinically relevant set of tools and strategies to support others with Parkinson's as their conversations begin to be impacted.

Interviews are widely used in qualitative research. However, interaction in this mode of data collection is limited to the participant and the interviewer (Kitzinger 1995). Focus groups facilitate group interaction and exchange of ideas among participants (Kitzinger 1995), especially when open communication about a common topic of interest is the goal (Morgan 1993). The interactional nature of focus groups helps participants to explore, build on, and enrich each other's ideas, and often yields more in‐depth information than a one‐on‐one interview (Murray et al. 1994). Focus groups facilitate natural interaction, as participants share examples from their lived experience that might not otherwise be explored (Kitzinger 1995) or may be considered sensitive (Guest et al. 2017). This research focussed on deepening current understanding of barriers, and particularly facilitators, to conversation. Focus groups offered the opportunity for participants to add detail and to topics raised by others and enrich understanding through the use of examples of experiences. This depth of information, combined with specific examples from the lived experience of people with Parkinson's and their partners, was the intended focus of the current study.

This study used focus groups to facilitate group interaction among people with Parkinson's and their communication partners to collect data on conversations from a larger number of participants than previous studies. The aims of the study were twofold: (i) to collect information about the perceptions of barriers to successful conversations from people with Parkinson's and their partners, and (ii) to collect information about strategies they used to support their conversations. This study used a strength‐based approach to identify the communication strategies developed as part of the lived experience of Parkinson's, with a view to informing the development of a Parkinson's conversation treatment program.

## 2 Method

This study employed a qualitative exploratory design. Five focus groups were used to determine the perceptions of barriers to successful conversations by people with Parkinson's and their partners, and the strategies they have employed that they perceived as helpful to support their conversations.

### 2.1 Recruitment

Approval was obtained from the ethics committee at the institution with which all authors are affiliated. Participants were recruited from an existing research database, support groups for people with Parkinson's, and private speech pathologists. Purposive sampling (criterion sampling) (Palinkas et al. 2015) was used to recruit participants who met the inclusion/exclusion criteria. Interested participants were given an information sheet and provided written consent.

### 2.2 Participants

Short ‘pre‐interviews’ with people indicating interest in the study enabled researchers to determine eligibility, while providing the means for participants to feel comfortable prior to participation in group settings (Jones et al. 2021). Author 1 completed pre‐interviews with interested participants via phone. Sharing sufficient information with participants prior to gaining consent also ensured participant understanding and engagement (Jones et al. 2021). Participants were people with Parkinson's and/or regular communication partners of people with Parkinson's who had difficulties with conversations. Both people with Parkinson's and their communication partners could participate independently. That is, a person with Parkinson's who wished to participate was not required to have a communication partner join the study.

People with Parkinson's who were included in the study (a) were 18 years or older; (b) had a self‐reported diagnosis of idiopathic Parkinson's disease (diagnosed by a General practitioner, Geriatrician, or Neurologist); (c) used English as their language of daily use; (d) had sufficient vision and hearing to participate in a focus group; (e) were able to functionally engage and concentrate for 60–90‐min and (e) self‐reported that they experienced difficulties in conversations (with their communication partner) arising from Parkinson's disease. People with Parkinson's were excluded if they (a) had a history of brain damage and (b) indicated they have a psychiatric diagnosis and/or behavioural disturbances. The telephone interview of cognitive status (TICS—M; de Jager et al. 2003) was used as a screen of cognitive status, and a TICS‐M score of 18 or less was used to exclude those with possible dementia

Five focus groups were conducted, involving 45 participants in total (25 people with Parkinson's and 20 communication partners). Sample size was decided iteratively based on data saturation. The communication partner was a partner (spouse, family member or friend) who (a) interacted with the person with Parkinson's at least three times a week, (b) experienced difficulties in conversations with the person with Parkinson's and (c) was not a paid carer. Table 1 provides participant demographic information. All except one communication partner was the spouse/partner of the person with Parkinson's. One dyad included the person with Parkinson's and their long‐term friend who lived with them.

**TABLE 1:** Summary of participant demographic information.

Focus group	Dyad number	Age (Years)				Duration of relationship (Years)	Time since diagnosis (Years)
		Person with Parkinson's	Gender	Communication partner	Gender		
1	1	76	F	81	M	55	6
1	2	77	M	71	F	51	6
2	3	71	M	69	F	42	3
2	4	57	M	56	F	32	4
2	5	72	F	74	M	54	4
3	6	74	M	69	F	53	9
3	7	83	M	83	F	64	6
4	8	78	M	76	F	57	5
4	9	72	M	68	F	14	11
4	10	87	M	80	F	53	3
4	11	71	M	57	M	20	2
4	12	78	F	79	M	57	10
4	13	83	M	82	F	65	5
4	14	77	M	69	F	50	6
4	15	83	M	78	F	60	19
4	16	80	F	83	M	62	6
4	17	77	M	76	F	44	9
4	18	69	M	79	F	44	1
4	19	78	M	76	F	57	8
4	20PwP	77	M				1
4	21PwP	68	F				1
4	20CP			76	F		
5	22PwP	78	M				1
5	23PwP	65	F				18
5	24PwP	76	M				1
5	25PwP	69	F				1
		m = 75.04	28%F vs. 72%M	m = 74.1	75%F vs. 25%M	m = 49.15	m = 5.84

*Note*: CP, communication partner; m, mean; PwP, people with Parkinson's.

### 2.3 Procedure

Focus groups were held at community centres, which helped create an equal, balanced environment in which to explore the experiences of participants. All focus groups were conducted by Author 1, who was not previously known to any participant. Each group was 90 min long. Focus groups were semi‐structured, and Author 1 used an interview guide as the basis of discussion (see Supporting Information). Focus group discussions explored (a) the perceived barriers to effective conversations when one person has Parkinson's and (b) the strategies used by both parties to facilitate effective conversations. A written form was handed over to each participant at the completion of each group, so that participants had the option to provide any information they were unable to disclose during the focus group. This could include information from reflections following the focus group or information they preferred to provide in a more confidential setting. No participants provided additional information outside of the focus groups.

Focus groups were audio and video recorded and transcribed verbatim by Author 1. A student assistant attended the group to take field notes, supporting the transcription process and reducing the risk of data loss.

Following data analysis, a summary of findings in plain language (in the form of a table listing strategies and barriers discussed) was shared with participants as a form of member checking (Motulsky 2021; Varpio et al. 2017). No additions were suggested, and participants who responded (*n* = 4) confirmed that the list captured their experience.

### 2.4 Trustworthiness and Rigour

To uphold the trustworthiness and rigour of the study's design and execution, the Consolidated Criteria for Reporting Qualitative Research (COREQ) checklist (Tong et al. 2007) was employed. Author 1 used reflective journaling and field notes to make assumptions explicit. This was then discussed with the wider research team to explore potential for bias (Elo et al. 2014; Elo and Kyngäs 2008).

### 2.5 Positionality and Reflexivity

The researchers adopted the role of collaborative stakeholders (‘non‐experts’) during the facilitation of focus groups. It is, however, acknowledged that the research team approached this study from a scientific and interventionist position. Academic team members were speech pathologists (Authors 1, 2 and 4) and a psychologist (Author 3) with experience in conducting research on topics related to Parkinson's disease.

### 2.6 Data Analysis

An inductive approach to Qualitative Content Analysis (QCA) (Elo and Kyngäs 2008) was used to identify the relevant categories and sub‐categories of the barriers to conversations and the strategies used by participants. The three stages described by Elo and Kyngäs (2008) were used to systematically collect, organise and analyse the data to abstract relevant categories.

#### 2.6.1 Preparation Phase

During the preparation phase, Author 1 engaged in a process of data immersion. Meaning units were defined by the team as statements, by participants or groups of participants, describing a barrier to effective conversations or a strategy used to support conversations.

#### 2.6.2 Organisation Phase

Author 1 completed open coding of the data using NVivo software (version 20.1.7.1). Coding and codes were reviewed collaboratively by the team at fortnightly meetings. Data saturation was achieved at the fifth focus group, with no new codes identified. To recognise when data saturation was achieved, data collection and analysis took place concurrently (Elo et al. 2014). Grouping data was completed by collapsing similar groups into broad, higher‐order related categories. This process involved comparing and contrasting data to identify group membership. The research team then came together to collaboratively categorise and abstract, with repeated refinements, until the final categories and sub‐categories were agreed upon.

#### 2.6.3 Reporting Phase

See Section 3.

## 3 Results

People with Parkinson's and their conversation partners described a range of barriers to effective conversations and a range of strategies they perceived helped their conversations. These are addressed separately and are summarised in Tables 2 and 3, respectively.

**TABLE 2:** Categories and sub‐categories related to barriers to successful conversations.

Category	Sub‐category
Barriers related to the person with Parkinson's	Changes to speech
	Changes to language
	Cognitive changes impact conversation
	Other Parkinson's symptoms impact conversation
	Age‐related changes
Barriers related to the communication partner	Natural communication style
	Response and feedback style
Barriers related to the conversation dynamic	Proximity
	Topic of conversation
	Group conversations
Background noise	
Limited information and services	Delay in access to services and information
	Lack of tools to manage changes

**TABLE 3:** Categories and sub‐categories related to strategies for conversations.

Category	Sub‐category	PwP	CP	Both	Explicit planning	In the moment
Strategies used in preparation for a conversation	Manage Parkinson's symptoms				x	
	Manage hearing difficulties				x	
	Manage fatigue				x	
	Manage environmental factors				x	
	Be aware of emotional readiness				x	
	Plan for group conversations				x	
Strategies used during a conversation	Use speech and voice strategies					x
	Use compensatory strategies to support speech					x
	Communicate clearly and succinctly					x
	Use prompts and feedback appropriately					x
	Show empathy towards speaker					x
	Get closer					x
	Use specific group conversation strategies				x	x
Strategies for phone use	Using technology				x	
Acceptance and awareness	Self‐acceptance and self‐awareness				x	
	Knowledge and understanding of others				x	
Services and information	Timeliness	n/a			n/a	
	Sufficient information	n/a			n/a	
Engage in activities	Everyday activities					x
	Therapeutic activities				x	

### 3.1 Barriers for Effective Conversations Experienced by People With Parkinson's and Their Communication Partners

Participants described five categories of barriers to conversation: barriers related to the person with Parkinson's, the communication partner, the conversation dynamic, background noise and barriers associated with limited information and services. The categorisation of barriers is presented in Table 2.

*Category 1: Barriers related to the person with Parkinson's*.

People with Parkinson's described how changes to their own abilities impacted conversation. These included (i) changes to speech, (ii) changes to language skills, (iii) factors affecting concentration and cognitive load, (iv) the impact of other Parkinson's symptoms on conversations and (v) age‐related changes.

*Sub‐category 1.1: Changes to speech*.

Changes to speech included increased slurring, monotonous speech, reduced breath support for speaking, reduced speech intelligibility and softer (reduced) volume. All of these barriers were described by all participants as impacting the ability of a person with Parkinson's to engage in conversations.

‘People keep saying I beg your pardon so obviously my pronunciation is getting affected’. (PwP24)

*Sub‐category 1.2: Changes to language skills*.

Most participants indicated that the language difficulties experienced by the person with Parkinson's impacted their ability to maintain a conversation. Language difficulties were described as challenges associated with the use of appropriate words to convey their message. This included word‐finding difficulties, being more literal in their thinking and using tangential communication.

‘If you're in a group, and there are three or four people and you want to say something, you've got to think of the words. And by the time you've thought of the words, the conversation's moved on’. (PwP22)

*Sub‐category 1.3: The impact of cognitive changes on conversations*.

All participants with Parkinson's identified that they had noted some level of cognitive changes related to their thinking skills and ability to concentrate. Participants reported that these difficulties negatively impacted their ability to have conversations, and that these difficulties were exacerbated by stress, fatigue and poor sleep quality.

‘I'm having lapses in concentration. Like I'm talking about one thing but then it's something else’. (PwP25)

*Sub‐category 1.4: The impact of other Parkinson's symptoms on conversations*.

Other symptoms of Parkinson's reportedly impacted some participants' ability to engage in conversations, including reduced self‐monitoring, excessive salivation and the effect of medication wearing off.

‘Sometimes with medication, the effect wears off, then that's harder for me to concentrate and communicate’. (PwP16)

*Sub‐category 1.5: Age‐related changes*.

Most participants described changes related to ageing, such as reduced memory and reduced hearing, which they felt exacerbated the effects of Parkinson's on their ability to effectively engage in conversation.

‘I have found that when she has said something, like “go and pick that cup,” but I have only heard “pick that,” and I don't hear “cup”’. (PwP18)

*Category 2: Barriers related to the communication partner*.

*Sub‐category 2.1: Natural communication style*.

The natural communication style of the partner was also noted as a barrier to successful conversations by most participants. The natural communication style was the way in which the partner had always communicated and reportedly often remained unchanged even after their partner had been diagnosed with Parkinson's. These included their fast rate of speech, providing too many options, or using responses that were not succinct.

‘Giving him too many options to choose from confuses him’. (CP11)

‘If someone waffles, I can't handle it’. (PwP4)

*Sub‐category 2.2: Response and feedback style*.

Several behaviours were seemingly used by the communication partner as a prompt or reminder, assuming it would support conversations. These included asking the person with Parkinson's to raise their voice or guessing what was said instead of clarifying. These were, however, described by some participants with Parkinson's as barriers to conversation as it hindered their interactions often making the person with Parkinson's angry or upset and required discussing this with their partners.

‘I always tell her raise your voice and she gets mad’. (CP1)

*Category 3: Barriers related to the conversation dynamic*.

All participants described barriers related to the inherent nature of conversations. This included the proximity of speakers, the topic of conversation, and challenges associated with group conversations.

*Sub‐category 3.1: Proximity*.

Proximity barriers included the distance between the speaker and listener and not looking at each other when talking. The inability to see each other meant they were unable to pick up on extra cues from facial expression and lip reading, which thereby impacted the conversation dynamic

‘You can't see the person to get a bit of extra cues off them’. (PwP23)

‘They are sitting far away but I wouldn't hear what they said….But I'll pick up a word they said and try to join in’. (PwP7)

*Sub‐category 3.2: Topic of conversation*.

A typical conversation involves discussing multiple topics with the topic changing frequently and sometimes very fast. These frequent and often fast topic changes were described as barriers to successful conversation. The causes of this difficulty were not explored in detail and may have been due to the partners changing topics frequently or cognitive changes experienced by the person with Parkinson's.

‘Sometimes in a conversation, we jump from one topic to another, but sometimes it's harder for him to get there so fast. When we're on the next topic he is still talking about the previous’. (CP8)

*Sub‐category 3.3: Group conversations*.

Group conversations were identified as much more challenging than one‐on‐one conversations, due to the tendency for people to talk simultaneously. People with Parkinson's reported that they often felt they were not given an opportunity to speak during group conversations.

‘I am not as confident in groups’. (PwP5)

*Category 4: Background noise*.

Background noise was reported by all participants to be an environmental factor that significantly exacerbated difficulties in conversations. Background noise was described as adding to the already existing challenges relating to thinking and concentration.

‘Any other background noise in general. Could be music, your phone or even the wind’. (PwP9)

*Category 5: Limited information and services*.

Many participants identified the limited information about Parkinson's and the limited services/support as impacting their ability to have successful conversations. Limited information and services made it difficult for people with Parkinson's and their families to access support to improve their conversations.

*Sub‐category 5.1: Delay in access to services and information*.

Most participants described how it took many years to get a diagnosis of Parkinson's. Such delays meant they were without support and Parkinson's‐specific information for long periods at a crucial time in their Parkinson's journey. In the pre‐diagnostic and early post‐diagnostic period, there was a distinct lack of information about the ‘dos and don'ts’ with regard to conversations.

‘I don't know the full extent of this, but I have to explain it to everyone else. That's quite a big psychological barrier because nowhere is there a “do I do this, or don't I do this” guide… what are the ramifications if I do this?’ (CP4)

*Sub‐category 5.2: Lack of tools to manage changes*.

Many participants reported they lacked the tools to manage changes to communication. Most participants reported difficulty maintaining speech therapy at home due to a lack of support and understanding about how the home/clinic speech therapy could be transferred into conversational settings.

‘It (speech therapy) helped in the therapy session, but it was hard at home’. (PwP9)

### 3.2 Strategies Used by People With Parkinson's and Their Partners That They Perceived Supported Conversations

Strategies described by participants to support conversations comprised six categories, including strategies they used in preparation for a conversation, strategies used during a conversation, strategies involving phone use, strategies associated with acceptance and awareness of Parkinson's, strategies related to services and information, and the importance of engaging in activities. Of the strategies described by participants, some related to the person with Parkinson's, some related to the communication partner, and some related to both parties in the conversations. Some strategies involved purposeful pre‐planning, while others were used more spontaneously in conversation. The categorisation of strategies is presented in Table 3.

*Category 1: Strategies used in preparation for a conversation*.

All participants described strategies they use to prepare for conversations, which would support them to interact more successfully. These included managing a range of factors such as Parkinson's symptoms, hearing difficulties, fatigue, environmental factors, emotional readiness and group settings.

*Sub‐category 1.1: Managing Parkinson's symptoms*.

All participants with Parkinson's described the importance of taking Parkinson's medication to support their communication skills. Medications supported conversations primarily by reducing the severity of their Parkinson's symptoms and alleviating difficulties managing saliva. Speech therapy was also described to assist in preparation for a conversation. For example, attending speech therapy and practising speech exercises helped some participants to increase their volume of voice during conversations.

‘I have been doing speech therapy. When I keep up with those exercises, I find that I'm louder’. (PwP6)

*Sub‐category 1.2: Managing hearing difficulties*.

Most participants described the importance of both the person with Parkinson's and the communication partner addressing any hearing issues to support their conversations. Taking a hearing test to rule out hearing difficulties, regularly testing and programming hearing aids, and always wearing prescribed hearing aids supported their overall conversations.

‘You need to regularly tune and program your hearing aid because it changes regularly’. (PwP12)

*Sub‐category 1.3: Manage fatigue*.

Fatigue significantly impacted conversations, with all participants describing the importance of timing of conversations to ensure the person with Parkinson's was less fatigued. This included making purposeful decisions, such as going out in the morning or before late evening, to reduce the risk of fatigue. Participants also suggested avoiding conversations when fatigued or taking a break from a conversation and re‐visiting it later.

‘If it doesn't work then I have given up trying and I'll excuse myself from the conversation’. (PwP6)

*Sub‐category 1.4 Manage environmental factors*.

Managing environmental factors, such as reducing background noise and removing distractions, assisted conversations by supporting the ability to concentrate on the conversation. All participants discussed the importance of making decisions to avoid places with background noise, choosing quiet places when going out, and sitting in the front and corner of venues for improved hearing. Removing distractions, such as turning the radio off during conversations in the car and turning the television/iPad off during conversations, also facilitated more successful conversations.

‘He's got a companion card so we can choose the corner seats. We don't get too in the middle … Make sure we are more on the outside. For access and easy hearing’. (CP2)

*Sub‐category 1.5: Awareness of emotional readiness*.

Some participants also discussed the importance of being emotionally ready to prepare for conversations. This included discussing with each other the challenges they experience when communicating, which helped both parties understand and empathise with the other(s) involved in the conversation. Participants also discussed the importance of managing the emotional lows and anxiety associated with engaging in conversation, to make sure they were ready to talk.

‘So we use strategies like taking them physically out of that place, like we go for a walk, or go to sleep. It kind of resets him and we can then talk’. (CP11)

*Sub‐category 1.6: Plan for group conversations*.

Most people with Parkinson's reported that group conversations were significantly more challenging than one‐on‐one conversations, as they involved multiple people and often comprised many different topics requiring attentional shifts. In preparation for group conversations, people with Parkinson's reported planning go‐to ‘conversation starters’ to facilitate the initiation of conversations. Participants also stated that being aware of the topic of conversation and finding a common topic of interest increased inclusion and engagement in conversations.

‘He has his toolbox of a set of questions to start a conversation. So he gets into a conversation without waiting for someone else to’. (CP4)

*Category 2: Strategies used during a conversation*.

*Sub‐category 2.1: Use speech and voice strategies*.

Strategies reported by all people with Parkinson's included consciously applying learned speech and voice strategies (such as intentionally raising one's voice), saying the words clearly and louder with added effort, taking a deep breath prior to speaking, and speaking more slowly.

‘I consciously think about it… that I need to make it louder’. (PwP3)

*Sub‐category 2.2: Use compensatory strategies beyond speech*.

Most participants discussed the importance of using compensatory strategies beyond speech to support conversation. This included taking notes of key points to keep track of the topic of conversation, lip‐reading, requesting clarification during conversation and requesting concise responses.

‘I must write it down so that I can go back to it’. (PwP1)

*Sub‐category 2.3: Communicate clearly and succinctly*.

One subcategory of strategies used by all communication partners was using specific and concise language to ensure the person with Parkinson's could easily understand. (e.g., ‘pass me the cup’ vs. ‘pass me that.’)

‘I must specify what it was’. (CP13)

*Sub‐category 2.4: Use prompts and feedback appropriately*.

Another subcategory of strategies reported by some communication partners related to the importance of providing appropriate prompts and sensitive feedback, which were carefully delivered. These included providing the person with Parkinson's gentle reminders about speech strategies and providing a word to prompt the person with Parkinson's to stay on‐topic during conversation.

‘Sometimes we can provide the right words and bring him back to where the conversation was’. (CP14)

*Sub‐category 2.5: Show empathy towards the speaker*.

Empathy was another subcategory of strategies, with most participants describing the need for understanding the challenges faced by the person with Parkinson's during conversations. Understanding of the communication challenges faced by the person with Parkinson's contributed to improved engagement in conversations.

‘It highlights the need for empathy in a conversation more than any’. (PwP3)

*Sub‐category 2.6: Get closer*.

Another subcategory of strategies used by all participants with Parkinson's and their communication partner related to physical proximity. By engaging in conversations in the same room and looking at each other when talking, participants were able to hear each other better and, consequently, engage in the conversation more successfully.

‘I have to literally go into the room where he is’. (CP15)

*Sub‐category 2.7: Use specific group conversation strategies*.

Several strategies for group conversations were discussed, and these related to the person with Parkinson's, the communication partner, or both parties. Strategies for the person with Parkinson's included checking the facial expressions of listeners to assess if they heard what was spoken and asking open‐ended questions so they can listen in a group rather than speak continually. This is because open‐ended questions allowed others to elaborate on their responses as opposed to short yes/no responses.

‘Be consciously thinking about listening rather than talking. And that works to integrate yourself back into the group’. (PwP3)

Some participants with Parkinson's reported that they often look at their partner to request support and to inform their partner that they needed to have a break from the conversation.

‘He quite often looks to me. So if he is losing a conversation somewhere… he'll look at me to help him’. (CP6)

One strategy used by the communication partner to support the person with Parkinson's in group conversations included getting the attention of the person with Parkinson's. Most communication partners described saying their name or an indirect prompt to initiate a conversation, improve the flow of the conversation, and/or bring their attention back to the conversation.

‘You have to say their name first to get their attention’. (CP13)

Intentionally giving the person with Parkinson's the opportunity to speak in a group further supported the person with Parkinson's to be included and engage in the conversation better.

‘It's almost like you want the people in the group, or the people you're talking to, to be aware that we want to say something, and say what do you think?’ (PwP22)

Strategies relevant to both the person with Parkinson's and the communication partner included limiting the number of people in the group conversation and talking about one topic at a time.

*Category 3: Strategies for phone use*.

All participants discussed strategies for using technology, specifically phone use. Strategies to support phone conversations included using video calls (as the person's face can be seen for additional cues), learning to talk to phone systems (especially with recorded messages), using ear pods or the speaker function, and using a stylus for text messages.

‘It's really good to be able to see them through Facetime’ (CP1)

The physical symptoms associated with Parkinson's, such as tremors, affected the ease with which people could use their phones. People with Parkinson's who had finger tremors reported that their ability to answer the phone or type text messages was impaired, reducing their conversational potential when using the phone.

*Category 4: Acceptance and awareness*.

The importance of acceptance and awareness of the changes associated with Parkinson's for oneself, as well as the knowledge and understanding of others were discussed by most participants as helping them with conversations.

*Sub‐category 4.1: Self‐acceptance and self‐awareness*.

Most people with Parkinson's discussed the importance of self‐acceptance of the change due to Parkinson's so that they could be comfortable telling people they have Parkinson's, subsequently enabling awareness of communication difficulties. Only with awareness of what is needed could people with Parkinson's and communication partners make the necessary changes to their conversations.

‘I start by telling people I have Parkinson's and then I think they understand’. (PwP16)

*Sub‐category 4.2: Knowledge and understanding of others*.

All participants also discussed the importance of knowledge and understanding about Parkinson's among other people. This included increased knowledge about Parkinson's among family members and medical professionals who can then provide specialised support.

‘Once they (family) realised what was wrong, what it's got, they became more understanding’. (PwP25)

*Category 5: Strategies related to services and information*.

*Sub‐category 5.1: Timeliness*.

All participants discussed the importance of timely diagnosis, medication, and timely therapy so that they could receive the required support to help them with conversations at the required time.

‘If we left the therapy any longer, it would have been difficult to carry on’. (CP4)

*Sub‐category 5.2: Sufficient information*.

Most participants highlighted the need for information about support services such as support groups, specialist nurses and therapy services to be readily available. This information would guide them to access the required information and support for conversation as the condition progressed.

‘There's a need at that very early point to have some kind of…. not therapy but some information’. (CP4)

*Category 6: Engaging in activities*.

All participants highlighted the importance of being involved in daily activities and engaging in general everyday conversations. This included two subcategories of activities.

*Sub‐category 6.1: Everyday activities*.

Engaging in everyday activities, such as being involved in daily chores and engaging in general conversations not related to Parkinson's, was raised. As important

‘It's important to try and keep the conversation normal. So not to allow the disability or the condition to take control. So going to a group where there was just a normal conversation gave him a real sense of worth to just talk to people. The more we can push for normality helps’. (CP11)

*Sub‐category 6.2: Therapeutic activities*.

The second subcategory of activities described by all participants was the importance of engaging in therapeutic activities such as regular exercise, singing and talking to children, to support general communication skills.

‘Kids are like one of the therapists’. (CP1)

## 4 Discussion

People with Parkinson's and their partners described their lived experience of the barriers to effective conversations and the strategies they employ to support more effective conversations. Findings from this study clearly demonstrate that effective conversations are dependent on the skills and behaviours of *all* those involved in the conversation, including the person with Parkinson's and their communication partner(s). Participants discussed a range of barriers to successful conversations, many of which highlighted the shared nature of these challenges and the contributions of both parties. Participants also described a range of strategies they use in everyday life to support them in having more successful conversations.

Many of the barriers to successful conversations discussed by participants reflected findings from previous research (Johansson et al. 2020; Miller et al. 2006; Whitehead 2010; Wylie et al. 2022). The types of barriers described included changes to the speech and language skills of the person with Parkinson's, behaviours of the communication partner related to their communication style, the way communication partners convey feedback, distance between speakers and background noise. These insights underscore the multifaceted nature of conversational difficulties in Parkinson's, highlighting the importance of addressing both individual, partner and environmental factors in communication support strategies (Wylie et al. 2022).

Findings from this study also aligned with previous research about what people with Parkinson's and their communication partners actively do to support conversations (Johansson et al. 2020; Wylie et al. 2022). These included the person with Parkinson's using speech and voice strategies, the communication partner modifying their communication style and the way they provide feedback, managing proximity between each other and managing background noise. Also similar to previous research (Johansson et al. 2020; Thilakaratne et al. 2021; Wylie et al. 2022), the findings of this study also suggest that conversations are jointly constructed and managed by both the person with Parkinson's and their communication partner. There is therefore a need for communication support to go beyond improving speech production for people with Parkinson's and include conversational interventions involving both parties (Thilakaratne et al. 2021; Wylie et al. 2022).

One particular finding that was unique to the present study was the impact of cognitive load on conversations. Participants reported that increased cognitive load adversely impacted the quality of conversations. Participants identified different factors that they felt added to the cognitive load associated with conversations. These factors included stress, fatigue, poor sleep quality, difficulties managing saliva and reduced self‐monitoring. Some participants indicated that whilst one factor in isolation would not significantly impact upon conversations, it was the combination of these factors that formed the significant barrier to having successful conversations. For example, engaging in a conversation when the person was fatigued and stressed made it difficult to focus and keep track of the topic of conversation.

Participants in the study also discussed how particular conversational dynamics influenced the success of conversations. Group conversations were described as much more challenging than one‐on‐one conversations. This finding may also relate to increased cognitive load. Compared to one‐on‐one conversations, group conversations require a person to process more information, attend to different speakers, and keep up with topic changes (Wylie et al. 2022). All of these demands involve working memory (Paas et al. 2003). Cognitive decline is a key feature of Parkinson's, and approximately 40% of those with Parkinson's meet the criteria for mild cognitive impairment (Baiano et al. 2020). Even minor difficulties with attention and working memory may impact upon cognitively demanding tasks, such as conversations (Whitworth et al. 1999). Although a general cognition screening tool was completed to exclude people with dementia (de Jager et al. 2003), this would not be able to identify any specific or subtle changes to cognition. Therefore, the participants in the present study may have had some mild cognitive issues that impacted their ability to engage in conversation.

Another novel finding from this research included the importance of anticipating problems and preparing strategies in advance of a conversation. These include strategies such as managing the timing of conversations in accordance with Parkinson's symptoms and fatigue, and managing proximity to each other based on environmental factors like background noise. The dyads involved in this study discussed the importance of forward planning a conversation to ensure the person with Parkinson's could engage and contribute. Preparing conversation starters and familiarising themselves with potential topics of conversation (such as making oneself familiar with an upcoming event/interaction) were strategies used by both parties to support the person with Parkinson's to engage in the conversation. These strategies involve higher‐order executive functions such as planning, an aspect of cognition that is commonly impacted in Parkinson's (Baiano et al. 2020).

While some strategies identified in this study involved planning, others were situationally determined and spontaneously implemented, which required some degree of self‐monitoring. Evidence suggests that people with Parkinson's do not accurately perceive or adjust their speech difficulties based on auditory feedback of their speech (Baiano et al. 2020). Self‐monitoring their speech difficulties and adjusting their speech accordingly is, therefore, a challenge in Parkinson's disease. People with Parkinson's reported that using open‐ended questions and listening/looking at their communication partner were intentional strategies that led to more effective conversations. Some participants adopted the role of an ‘active listener’ in conversations. This involved behaviours such as maintaining eye contact, head nodding and using conversation fillers such as ‘mm hmm’ and ‘yeah’, to help them feel included and engaged. Communication partners also used intentional strategies during conversations, such as providing indirect or subtle prompts to remind the person with Parkinson's of the topic of conversation or inviting the person with Parkinson's to speak to promote their involvement in conversation. This support from communication partners helps to increase the opportunities for their partner to participate in conversations, and is increasingly used in interventions for a range of other types of communication disorders (Eriksson et al. 2016; Simmons‐Mackie et al. 2016).

Another finding unique to this study that participants highlighted was the importance of making an effort to continue to engage in everyday activities and participating in general conversations, as well as activities that have a general therapeutic effect, such as physical exercise. Engaging in these activities helped people with Parkinson's to re‐direct their attention to focus on everyday life and gave them topics to talk about (Miller et al. 2006). Finally, timely diagnosis, appropriate medication and access to therapy were identified as key Parkinson's‐specific factors that supported effective communication. Participants emphasised that acceptance and awareness of the condition, combined with informed family members and knowledgeable healthcare professionals, played a crucial role in facilitating early diagnosis and prompt referral to speech and language therapy. This, in turn, enabled individuals with Parkinson's and their families to access communication support at a stage when it could be most impactful (Miller 2017; Miller et al. 2006).

## 5 Implications of New Findings

The value of this research study lies in the exploration of strategies perceived as effective in supporting conversational success by people with Parkinson's and their communication partners. While several studies have explored the barriers to conversation for people with Parkinson's (Johansson et al. 2020; Whitehead 2010), this research explicitly identifies strategies reportedly used by people with Parkinson's to support conversational success, contributing to a growing evidence base of ‘what works’ grounded in lived experiences. While some previous work has explored conversational strategies (Wylie et al. 2022), this work adds detail about the nature of these strategies. Findings, including strategies for explicit planning of conversations, add new information to existing literature. Given the potential impact of changes in cognitive function, it is clear that interventions involving strategy use must be customised to the cognitive functioning of both the person with Parkinson's and their communication partner and reduce the cognitive load as much as possible for all involved. This lived experience evidence offers the potential to further inform the content of conversation interventions that incorporate both the person with Parkinson's and their communication partner, such as those proposed by Clay et al. (2023), to support people with Parkinson's and their partners to have more effective conversations.

## 6 Limitations and Future Research

Whilst the present findings suggest that cognition impacts upon conversation success, cognitive functioning was not assessed in any detail. A cognitive screen was completed to ensure people with significant cognitive difficulties were not included. Future research should explore in more detail how cognitive functioning and cognitive load interact and impact conversations for people with Parkinson's and their communication partners. Future research could also explore the strategies employed in conversations by communication partners to compensate for changes in cognitive functioning in the person with Parkinson's. Information such as the index of multiple deprivation and a formal and in‐depth communication assessment to assess the severity of dysarthria could not be completed with each participant, and therefore their experiences of difficulties in conversation were self‐reported. Future research could objectively measure the quality of conversations and try to qualify the effectiveness of strategies against agreed‐upon outcomes.

## 7 Conclusion

The current study describes the barriers faced by people with Parkinson's and their communication partners during conversations and the strategies they use to support their conversations. This study provides a unique insight into communication issues that are not typically discussed in depth. The large sample in this study and the use of focus groups facilitated in‐depth discussions, which resulted in rich data that was not previously reported. Findings from this study align with the findings of Wylie et al. (2022) and Johansson et al. (2020), which identify a range of barriers that impact conversations for people with Parkinson's. These relate to the person with Parkinson's, the communication partner, the conversation dynamic, and the conversation setting. This study adds new findings related to the impact of cognition and cognitive load on conversations and describes how barriers associated with limited information and services impact conversations.

Participants in this study described their lived experience of Parkinson's and shared the strategies they used to support conversations. These included strategies that could be used in preparation for and during conversations, with phone use, strategies related to acceptance and awareness of the condition and accessing services and information. This work highlights the important role played by both the person with Parkinson's and their communication partner in promoting conversational success to support their quality of life. It also highlights the need for future research assessing the effectiveness of conversation strategies that people with Parkinson's and their communication partners perceive to be effective.

## Funding

This study forms part of the first author's doctoral research program supported by an Australian Government Research Training Program Scholarship.

## Ethics Statement

Ethics approval for this study was granted by the Curtin University Human Research Ethics Committee (HRE2023‐0468) and Parkinson's Western Australia.

## Conflicts of Interest

The authors declare no conflicts of interest.

## Supporting information




**Supporting File**: jlcd70202‐sup‐0001‐SuppMat.docx

## Data Availability

The data that support the findings of this study are available on request from the corresponding author. The data are not publicly available due to privacy or ethical restrictions.
