# Supplementary material for: Conversation Barriers and Strategies Used by People With Parkinson's and Their Partners to Support Conversation
Source: Int J Lang Commun Disord. 2026 Jan 25;61(2):e70202. doi: 10.1111/1460-6984.70202 (PMC12833553; doi:10.1111/1460-6984.70202)
Supplement: Supplementary file 1 — Supporting File: jlcd70202‐sup‐0001‐SuppMat.docx [file JLCD-61-0-s001.docx]

**Semi-structured interview topic guide for focus groups**

| **Session will begin with rapport building activities and discussion of group rules.** | |
| --- | --- |
| **Topic 1: Barriers faced during conversations** | **What are some of the barriers or challenges you face when having conversations with people?**  Probes:  Do you find that the changes in your speech can be challenging?  What aspects about your speech can be challenging?  Do you feel you receive sufficient support from the listener to help you converse with them?  Are there any ways the people you're taking to can make it more challenging?  What other factors hinder you from continuing your conversations with people? |
|  | **Think of an incident when it was very difficult to have a conversation with someone.**  Probes:  What were some of the reasons why this was difficult?  Were you able to address these difficulties?  How did you do so?  Did anyone else help you address these difficulties? |
| **Topic 2: Strategies used to support conversations** | **What are some of the strategies you use regularly to support your conversations?**  Probes:  Are there any techniques you use to make your speech sound clearer?  What other strategies do you use to help the listener understand you better?  Do other people use any strategies that support you to have a better conversation with them?  What advice do you have for people who talk to you? How can they help you communicate better?  What other factors would help you have a good conversation? |
|  | **Think of an incident when it was very easy to have a conversation with someone.**  Probes:  What were some of the reasons why this was easy and successful?  Were there any specific strategies you used here?  Were there any strategies you used before this incident that supported you?  Did anyone else involved use strategies that also supported the conversation? |
|  | **How would you describe an ideal situation when you have a good conversation with someone?**  Probes:  Is there anything you do already to achieve this?  Is there anything your family members and friends do to achieve this?  Are there any other modifications you make to achieve this? |
| **Topic 5: Other information** | **Is there anything else you wanted to comment on that we have not covered?**  **Or anything that you did not get a chance to discuss that you would like to mention?** |
